# Oxytocin in Pregnancy and the Postpartum: Relations to Labor and Its Management

**DOI:** 10.3389/fpubh.2014.00001

**Published:** 2014-01-27

**Authors:** Marie Prevost, Phyllis Zelkowitz, Togas Tulandi, Barbara Hayton, Nancy Feeley, C. Sue Carter, Lawrence Joseph, Hossein Pournajafi-Nazarloo, Erin Yong Ping, Haim Abenhaim, Ian Gold

**Affiliations:** ^1^Departments of Psychiatry and Philosophy, McGill University, Montreal, QC, Canada; ^2^Lady Davis Institute, Jewish General Hospital, McGill University, Montreal, QC, Canada; ^3^Department of Obstetrics and Gynecology, Jewish General Hospital, McGill University, Montreal, QC, Canada; ^4^Department of Psychiatry, University of North Carolina, Chapel Hill, NC, USA; ^5^Division of Clinical Epidemiology, McGill University, Montreal, QC, Canada

**Keywords:** labor, oxytocin, pregnancy, epidural, syntocinon

## Abstract

The purpose of this study was to examine variations in endogenous oxytocin levels in pregnancy and postpartum state. We also explored the associations between delivery variables and oxytocin levels. A final sample of 272 mothers in their first trimester of pregnancy was included for the study. Blood samples were drawn during the first trimester and third trimester of pregnancy and at 8 weeks postpartum. Socio-demographic data were collected at each time point and medical files were consulted for delivery details. In most women, levels of circulating oxytocin increased from the first to third trimester of pregnancy followed by a decrease in the postpartum period. Oxytocin levels varied considerably between individuals, ranging from 50 pg/mL to over 2000 pg/mL. Parity was the main predictor of oxytocin levels in the third trimester of pregnancy and of oxytocin level changes from the first to the third trimester of pregnancy. Oxytocin levels in the third trimester of pregnancy predicted a self-reported negative labor experience and increased the chances of having an epidural. Intrapartum exogenous oxytocin was positively associated with levels of oxytocin during the postpartum period. Our exploratory results suggest that circulating oxytocin levels during the third trimester of pregnancy may predict the type of labor a woman will experience. More importantly, the quantity of intrapartum exogenous oxytocin administered during labor predicted plasma oxytocin levels 2 months postpartum, suggesting a possible long-term effect of this routine intervention, the consequences of which are largely unknown.

## Introduction

Pregnancy is a unique state that is accompanied by numerous biological and psychological changes. While there are known changes in the levels of hormones such as progesterone and estrogen in pregnant women (1), there is a paucity of information about variations in levels of oxytocin, crucial to parturition and lactation. Oxytocin is a neuropeptide that is synthesized centrally in the paraventricular and supraoptic nuclei of the hypothalamus and is released into the bloodstream via the posterior pituitary during labor, lactation, uterine dilatation, stress, after sexual stimulation, and possibly during different types of social interactions (2–5). Oxytocin is also released centrally in other parts of the brain (6) and is synthesized peripherally in the ovary, testis, adrenal, thymus, and pancreas (3).

In non-human primates, no reliable pattern of variation of oxytocin during pregnancy or the postpartum period has been observed, but a fourfold increase on the day of delivery has been reported (7). A study in cows showed a similar peak of plasma oxytocin levels on the day of delivery but very stable and low levels of circulating oxytocin during the late pregnancy period (8).

In humans, different methods for measuring oxytocin during pregnancy and labor have been used including radioimmunoassay and enzyme immunoassay, and a variety of results have been reported. Some studies have found that oxytocin levels increase at the onset of labor and during labor compared to 1 or 2 weeks before labor (9), reaching a peak just when the head of the baby is delivered (10). More than two decades ago, Dawood (11) reviewed the literature and concluded that plasma oxytocin levels increase during pregnancy and parturition. de Geest and colleagues (12) reported an increase of oxytocin levels with a maximum at term. In another study, levels of oxytocin were found to increase slowly until delivery and then decrease up to 8 weeks postpartum (13). One group reported higher levels of oxytocin at 36 weeks of pregnancy than 1 day after delivery or later (14). Another group observed a steady increase in plasma levels of the oxytocin precursor, neurophysin–oxytocin, during pregnancy followed by a decrease between 7 and 42 days postpartum but stable plasma oxytocin levels during pregnancy (15). In contrast, one team recently reported no variations of oxytocin levels between the first trimester, the third trimester, and the first month postpartum (16, 17). Fuchs and Fuchs (1) noted that plasma levels of oxytocin can be difficult to measure because oxytocin has a half-life of a few minutes, is found in low concentrations compared to other hormones, and is released in a pulsatile pattern. These factors, as well as the differences in experimental methodology, could explain the great variation of results in the oxytocin literature.

Given the lack of clarity concerning oxytocin in pregnancy and the postpartum period, the present longitudinal study aimed (a) to describe the range of oxytocin levels in a larger sample of pregnant women than had been studied hitherto; (b) to estimate plasma oxytocin variations during pregnancy and the postpartum period; and (c) to explore the relation between oxytocin levels and labor outcomes. Thus, our main objective was to describe how plasma oxytocin levels vary in a large cohort of pregnant women. Our secondary interest was to raise, or confirm, hypotheses about oxytocin correlates by exploring its association with a number of labor variables available in our cohort.

## Materials and Methods

### Participants

A total of 272 women were tested. We recruited 342 pregnant women during their first trimester of pregnancy at an obstetrics clinic in a general hospital (*n* = 245) and in a birthing center (*n* = 97), in Montreal. Inclusion criteria were: pregnancy with a single fetus; between 10 and 15 weeks of pregnancy; speaking and reading either English or French. Exclusion criteria were: younger than 18. Eleven women miscarried, 7 moved out of Montreal, 32 dropped out (including 6 for health reasons), 6 were excluded for health reasons, 13 had unreliable test results, and 1 was enrolled twice. Oxytocin levels were therefore obtained at three time points in the final sample of 272 women: during the first trimester (T1), the third trimester (T2), and 2 months postpartum (T3).

### Procedure

The study was approved by the Jewish General Hospital Research Ethics Committee and the Maison de Naissance Côtes des Neiges. A recruiter approached women during their first prenatal visit or during a general information session. Women meeting all of the inclusion criteria and willing to take part in the study gave written informed consent and were tested the same day. A second meeting took place during their third trimester of pregnancy, and a third meeting took place at the mothers’ homes 8 weeks postpartum. At each meeting, women completed a series of questionnaires described below in English or French, and blood samples (10 mL) were then drawn by a nurse or a midwife. Women received $25 per visit. Blood samples were drawn between 9 a.m. and 5 p.m., as soon as the nurse was available or as soon as the meeting with the doctor was over. At the postpartum session, if the mother was breastfeeding or bottle-feeding when the research assistant and nurse arrived, blood samples were drawn at least 30 min after the completion of feeding. Data were collected between July 2009 and September 2011.

### Data collection

#### Medical chart variables

Data were extracted from participants’ medical charts. Variables included: length of pregnancy; use of an epidural anesthesia; quantity of intrapartum exogenous oxytocin administered (for induction or augmentation of labor), for mothers who gave birth at the hospital; route of delivery (vaginal or cesarean); and duration of labor. The type of cesarean delivery was classified, according to Lucas and colleagues’ definition (18), in one of four categories: emergency, urgent, scheduled, and elective. Duration of labor was calculated from the start of the active phase, defined as 3 cm dilation and/or contraction every 3–5 min.

#### Socio-demographic variables

Data about age, education level, marital status, parity, and previous miscarriages were collected from a self-report questionnaire completed by every participant at her first visit.

#### Somatization

Here, somatization is defined as the experience of physical symptoms most likely due to mental factors. One hypothesis is that oxytocin might modulate the link between these mental factors and their physical symptoms through its influence on the immune system, observed in humans (19) and in animals (20, 21). We used the 12 items of the somatization subscale of the SCL-90-R (22). Participants had to rate the presence of various symptoms such as headaches, soreness in muscles, heavy arms or legs, or dizziness on a 5-point Likert scale (from “not at all” to “extremely”). This scale has a good internal consistency (α = 0.86).

#### Experience of labor

An interview, conducted through phone at about 3 weeks postpartum (mean = 26 days, SD = 15), evaluated whether the women had a very negative, somewhat negative, neither negative nor positive, somewhat positive, or very positive labor experience.

#### Breastfeeding

Since oxytocin is known to play a role in milk ejection during lactation (23), we asked mothers whether they were still breastfeeding at 2 months postpartum in order to explore whether plasma oxytocin levels were associated with breastfeeding.

### Blood sample analysis

Blood was collected from participants in a heparinized tube, stored on ice until it could be centrifuged at 1600 × *g* for 15 min at 4°C. The plasma was frozen at −80°C. The samples were divided into two aliquots. One was sent to the laboratory of Sue Carter, then at the University of Illinois in Chicago for analysis; the second was stored for future testing.

Oxytocin levels were measured using a commercially available enzyme-linked immunosorbent assay (EIA: Enzo Life Sciences Inc.). Direct measurement was performed following the manufacturer’s protocols and samples were diluted 1:2 or 1:4 as described previously (24, 25). Samples were not extracted prior to analysis and all samples were run in duplicate or triplicate. The minimum detection limit for oxytocin was 15.6 pg/mL and inter and intra assay coefficients of variability were less than 8.7%.

### Data analysis

We first compiled descriptive statistics for all variables collected in the study, including means, standard deviations, medians, and inter-quartile ranges and proportions, as appropriate. We next calculated correlation coefficients to examine, which variables were associated with oxytocin, and with each other, to search for possible confounding variables. All variables associated with oxytocin levels were included in the models exploring the relationship between oxytocin levels and labor or postpartum variables.

Three oxytocin measurements were taken for each participant. It is important to note that previous research has suggested that oxytocin levels measured in three repeated blood samples collected from 65 participants at intervals of 5 min showed greater inter-individual variation than intra-individual variation, with an intraclass correlation coefficient suggesting modest reliability (personal communication from Elizabeth Hoge). The intraclass correlation coefficient indicates how much agreement there is between two or more repeated measures. Even though this agreement is moderate (26) in the case of oxytocin, it remains greater than the variation between participants. As there was considerable between-subject variability in the trajectories, we fit a Bayesian random effects hierarchical model to these data. This model allows individual-level estimation of each woman’s trajectory, while at the same time estimating and summarizing overall trends across women. At the first level of the hierarchical model, each woman’s oxytocin values followed a piecewise linear regression model, with distinct parameters for the slopes from time 1 to time 2 and from time 2 to time 3. Therefore, for each woman *i*, *i* = I, 2, …, 272, three parameters were estimated: an intercept α*_i_* representing the baseline value at time 1, a first slope parameter β_1_*_i_* representing the average change from time 1 to time 2, and a second slope β_2_*_i_* representing the average change from time 2 to time 3. At the second level of this model, the three parameters from the first level are each assumed to follow normal densities across subjects. The mean of each normal density represents the average parameter values across all women (average intercept and average slopes), while the standard deviation of the normal distribution provides the between-subject variability in the parameter values across women. Finally, the third level of the hierarchical model placed diffuse prior distributions across all parameters from the second level, ensuring that the data drive all inferences.

Linear and logistic regression models were used to estimate the best predictors of oxytocin levels at each time point, and which outcomes oxytocin levels predicted. We included the following variables: parity, location of delivery, transfer from the birth center to the hospital, epidural, duration of labor, categories of cesarean birth, somatoform symptoms, positive experience of labor, negative experience of labor, intrapartum exogenous oxytocin administered, breastfeeding at 2 months postpartum, change of oxytocin levels from the first to the third trimester, oxytocin levels during the first trimester, during the third trimester, and during the postpartum period. We ran several models, as displayed in Table 3. The outcome variables were: levels of oxytocin at the third trimester of pregnancy, oxytocin levels changes from first to third trimester, labor duration, epidural, quantity of intrapartum oxytocin administered, negative experience of labor, positive experience of labor, breastfeeding, and oxytocin levels at 2 months postpartum. While several models including all the variables mentioned above were run for each outcome in order to investigate possible confounding, we present only the model that includes important predictors or confounders. This selection of models was based on clinical judgment. As such, if the variable has no appreciable effect and was not a confounder, it was omitted, otherwise it was included. More specifically, for each outcome estimated, we report here in Table 3 only the model that was optimized to make future predictions about the outcome. When oxytocin is the outcome, all other variables excluding oxytocin levels were included. When labor variables were estimated, all other variables and oxytocin levels were included. It is worth noting that we did not correct our confidence interval levels for multiple comparisons even though we estimated many parameters. Due to the exploratory nature of these analyses, we prefer not to risk eliminating any potential valid result.

The longitudinal models were fit using WinBUGS software (version 1.4.3, MRC Biostatistics Unit, Cambridge, UK). Other models were run using R (version 2.12, R Development Core Team, 2012).

## Results

### Descriptive statistics

Descriptive statistics are shown in Table 1. Most participants were married or living with a partner; had a vaginal delivery; had not experienced a previous miscarriage; and delivered around 39.6 weeks of gestation (SD = 1.3). Most were pregnant with their first or second child. Seven women delivered pre-term, and one baby did not survive. These women were later excluded from the study. The mean gestational age was 11.9 weeks (SD = 2.1) at T1 and 32.5 weeks (SD = 2.0) at T2.

**Table 1:** **Descriptive statistics of the participants**.

	Mean (standard deviation)
Age (years)	31.6 (4.6)
Education (years)	15.3 (2.6)
Length of pregnancy (weeks, *n* = 271)	39.6 (1.2)
Duration of labor (h, *n* = 232)	8.6 (6.7)

**Marital status (*N* = 341)**	**Percentage**

Single	4.4
Married	68.0
Living with partner	25.4
Divorced/separated	2.2

**Parity**

0	47
1	38
2	10
3 or +	5

**Type of delivery**

Vaginal	74
Vaginal after cesarean birth	4
Elective cesarean birth^a^	9
Scheduled cesarean birth^a^	7
Urgent cesarean birth^a^	5
Emergency cesarean birth^a^	1

**Miscarriage**

0	71
1	24
2 or +	5

**N* = 272 unless otherwise specified*.

*^a^Cesarean births are classified using Lucas et al.’s criteria (18)*.

### Range and variation of oxytocin levels

**Figure 1:**
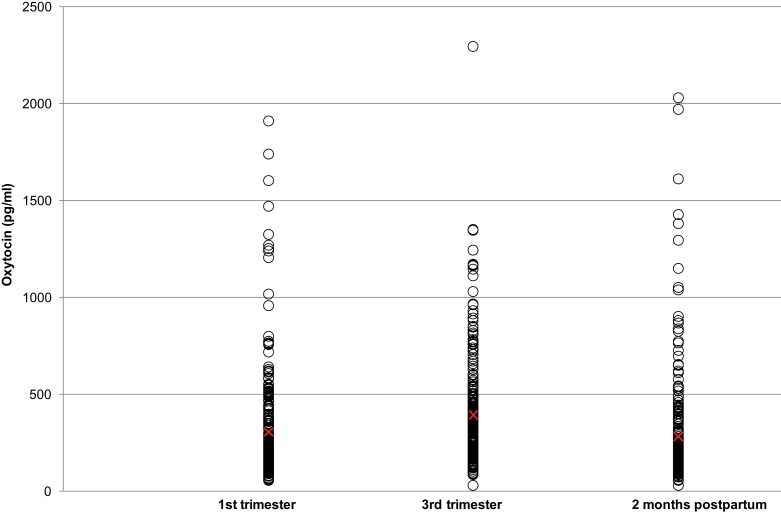
**Plasma oxytocin levels during the pregnancy and postpartum periods**. Red crosses indicate mean values for each time period.

Figure 1 shows that oxytocin levels varied considerably across individuals (Min = 32.3 pg/mL; Max = 2297.6 pg/mL). The mean oxytocin levels were 308.3 pg/mL (SD = 272.3) at T1; 396.5 pg/mL (SD = 278.8) at T2; and 286.3 pg/mL (SD = 272.7) at T3. Intra-individual mean variations from T1 to T2 were 88.1 pg/mL (SD = 223.6) and from T1 to T3 were −22.1 pg/mL (SD = 170.3). As there were no differences in oxytocin levels between centers [mean difference T1: −10.5 pg/mL, CI = (−80.6; 59.5); mean difference T2: −16.1 pg/mL, CI = (−87.8, 55.5); mean difference T3: 44.6 pg/mL, CI = (−25.3, 114.5)], data are presented for the whole group instead of by center.

**Figure 2:**
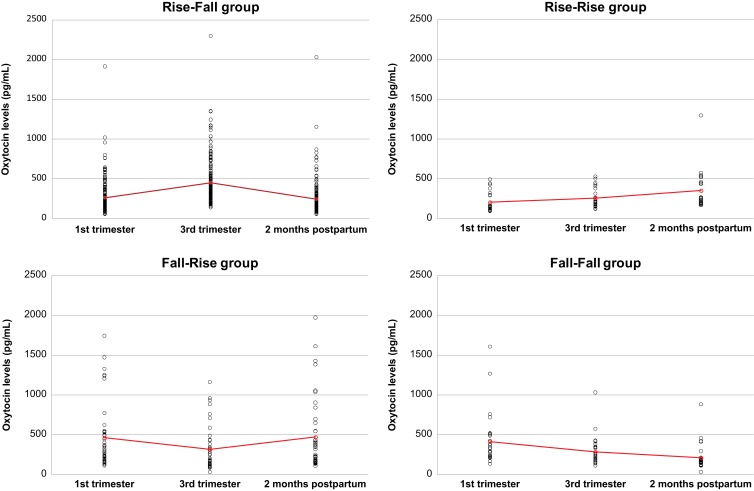
**Plasma oxytocin levels in the four groups of women with different patterns of oxytocin level variations**. Red crosses indicate mean values for each time period.

Our hierarchical model estimated that oxytocin levels increased from T1 to T2 [change of 0.60 pg/mL estimated per day, CI = (0.41; 0.79)] and decreased from T2 to T3 [change of −1.03 pg/mL estimated per day, CI = (−1.30; −0.76)]. The standard deviations of the slopes between T1 and T2 and between T2 and T3 were very large [T1–T2: mean SD = 1.57, CI = (1.45; 1.71); T2–T3: mean SD = 2.26, CI = (2.07; 2.46)]. This suggests that this pattern of positive slope from T1 to T2 and negative slope from T2 to T3 was not followed by all women. Indeed, as shown in Figure 2, 64.7% of the mothers had rising oxytocin levels from T1 to T2 and falling levels from T2 to T3 (group Rise–Fall); 8.5% had rising levels from T1 to T2 as well as from T2 to T3 (group Rise–Rise); 15.4% had falling levels from T1 to T2 and increasing levels from T2 to T3 (group Fall–Rise); and 11.4% had decreasing levels from T1 to T2 as well as T2 to T3 (group Fall–Fall).

### Relationship between oxytocin levels and labor outcomes

The four groups defined above were compared for labor duration, somatoform symptoms, intrapartum exogenous oxytocin, and experience of labor, parity, and route of delivery (Table 2). Labor was shorter in the Fall–Rise group than in the Rise–Fall or Rise–Rise groups [mean difference of 2.37 h, 95% CI = (0.73; 4.01) and of 4.23 h, CI = (0.76; 7.70), respectively]. The Fall–Fall group experienced more somatoforms symptoms at time 2 than the Rise–Fall group [mean difference of 1.27, CI (0.29; 2.20)] and received more intrapartum exogenous oxytocin than the Fall–Rise group [mean difference of 2.04 IU, CI = (0.08; 4.81)]. Finally, more mothers from the Fall–Rise group experienced a very positive or somewhat positive labor than mothers in the Fall–Fall or Rise–Fall groups [mean difference of 26.5%, CI = (6.7; 46.4) and of 15.2%, CI = (3.2; 27.2), respectively].

**Table 2:** **Descriptive statistics for each group of women according to their pattern of oxytocin level variation**.

Continuous variables	Rise–rise group			Rise–fall group			Fall–rise group			Fall–fall group		
	*N*	Mean	SD	*N*	Mean	SD	*N*	Mean	SD	*N*	Mean	SD
Labor duration (h)	18	10.51	6.48	153	8.65	5.24	32	6.28	3.96	26	9.29	7.01
Somatoform symptoms T1	23	4.96	3.08	176	5.28	2.80	42	4.81	2.17	31	5.61	2.69
Somatoform symptoms T2	23	5.74	2.77	175	5.73	2.67	40	6.50	2.74	31	6.97	2.39
Exogenous oxytocin (IU)	23	1.90	4.02	175	1.23	2.52	42	0.58	1.85	31	3.02	6.29

**Dichotomous variables**	***N***	**Percentage**		***N***	**Percentage**		***N***	**Percentage**		***N***	**Percentage**	

Cesarean birth	23	13.0		175	5.1		42	4.8		31	6.5	
Epidural	23	60.9		175	61.7		42	45.2		31	51.6	
Negative experience labor	22	13.6		175	11.4		41	4.9		31	19.4	
Positive experience labor	22	81.8		175	72.6		41	87.8		31	61.3	
First child	23	43.5		176	51.1		42	66.7		31	48.4	

*T1 = first trimester of pregnancy; T2 = third trimester of pregnancy, T3 = 2 months postpartum. Rise–rise = oxytocin levels rise from T1 to T2 and from T2 to T3; rise–fall = oxytocin levels rise from T1 to T2 and fall from T2 to T3; fall–rise = oxytocin levels fall from T1 to T2 and rise from T2 to T3; fall–fall = oxytocin levels fall from T1 to T2 and from T2 to T3*.

To explore the associations between oxytocin levels and the other variables, we ran a series of multivariate models, summarized in Table 3 and described below.

**Table 3:** **Relationship between oxytocin levels and the variables of interest**.

**VARIABLES PREDICTING OXYTOCIN LEVELS**				
**Oxytocin change T1 to T2**	Parity			
	−33.853 (−59.345, −8.360)			

**Oxytocin T2**	Parity			

	−29.943 (−62.745, 2.860)			

**Oxytocin T3**	Exogenous oxytocin			

	15.592 (5.733, 25.452)			

**VARIABLES PREDICTING LABOR**				
**Exogenous oxytocin**	Epidural	Parity		

	1.787 (1.038, 2.535)	−0.529 (−0.890, −0.168)		

**Labor duration**	Parity	Center	Exogenous oxytocin	Transfer to hospital

	−1.456 (−2.021, −0.891)	−4.417 (−5.8525, −3.009)	0.598 (0.430, 0.766)	3.985 (1.990, 5.979)

**Negative experience of labor^a^**	Cesarean birth	Exogenous oxytocin	Oxytocin T2	

	17.813 (4.448, 71.336)	1.296 (1.132, 1.484)	1.002 (1.000, 1.003)	

**Positive experience of labor^a^**	Cesarean birth	Exogenous oxytocin		

	0.790 (0.701, 0.889)	0.081 (0.020, 0.323)		

**Epidural^a^**	Center	Exogenous oxytocin	Oxytocin T1	Oxytocin T2

	0.116 (0.061, 0.223)	1.478 (1.198, 1.824)	0.997 (0.995, 0.999)	1.003 (1.001, 1.004)

**Epidural^a^**	Center	Exogenous oxytocin	Oxytocin change T1 to T2	

	0.117 (0.061, 0.225)	1.456 (1.181, 1.795)	1.003 (1.001, 1.004)	

**VARIABLES PREDICTING BREASTFEEDING AT T3**				
**Breastfeeding^a^**	Center			

	7.653 (1.778, 32.940)			

*Outcome (dependent variable) is indicated in bold. For each outcome, all other variables were included in the analyses and only the model optimized for future predictions about the outcome is reported. For continuous outcomes, the parameter estimates provide the change in outcome for a one unit change in the independent variable, with 95% confidence interval in brackets. When the outcome is a dichotomous variable (indicated by a), we provide the odds ratio for a one unit change, with the confidence interval in brackets. T1 = first trimester of pregnancy, T2 = third trimester of pregnancy, T3 = 2 months postpartum*.

Oxytocin levels at the third trimester of pregnancy and changes of oxytocin levels from the first to third trimester of pregnancy, were predicted by parity, with greater parity predicting lower oxytocin and a smaller variation in oxytocin between the first and third trimester. Postpartum oxytocin levels (T3) were predicted by the quantity of intrapartum exogenous oxytocin received, with more intrapartum oxytocin predicting higher plasma oxytocin levels at 2 months postpartum. There were no other significant predictors of plasma oxytocin levels at T3.

A “very negative” or “somewhat negative” labor was predicted by oxytocin at the third trimester, the type of cesarean delivery, and the quantity of intrapartum oxytocin received. In contrast, only intrapartum exogenous oxytocin and cesarean birth type were relevant variables explaining positive labor scores. Having an epidural was predicted by oxytocin levels at the first and third trimester (or oxytocin changes from first to third trimester), the birth location (hospital or midwife clinic), and the quantity of intrapartum exogenous oxytocin received.

### Relationship between oxytocin and breastfeeding

Only 30 women had stopped breastfeeding at 2 months postpartum. They stopped breastfeeding at around 7 weeks postpartum (*n* = 13, data were not collected for the others mothers). Mean postpartum oxytocin levels for the mothers who did not, or had stopped, breastfeeding were 327.9 pg/mL (SD = 187.0), and 284.9 pg/mL (SD = 285.7) for the women who were still breastfeeding. Using a Wilcoxon method, the mean estimated difference between the two groups was 62.5 pg/mL [CI = (15.8; 117.1)]. Using a multivariate model to test, which variables predicted breastfeeding, we found that oxytocin levels were not good predictors of breastfeeding at T3. The only variable that was associated with breastfeeding was location of delivery, as reported in Table 3. Indeed, all the women who stopped breastfeeding gave birth at the hospital.

## Discussion

We measured plasma oxytocin levels in healthy women at three time points: during the first and third trimesters of pregnancy and 2 months postpartum. There were large inter-individual differences in oxytocin levels, with some women having less than 50 pg/mL and others having more than 2000 pg/mL (Figure 1). For most women, oxytocin levels rose from the first to the third trimester and fell during the postpartum period. These observations are in accordance with previous studies (12, 13). However, one team has reported stable oxytocin levels across pregnancy and 1 month after delivery (16, 17). To the best of our knowledge, our study is the first to investigate oxytocin levels during pregnancy in a sample of this size.

Animal studies suggest that oxytocin levels rise as delivery approaches, in part because oxytocin has a contractile function on the uterine muscles (1), and its release is triggered by cervical stimulation (27). In addition, the number of oxytocin receptors increases dramatically in late pregnancy (28, 29). Based on these findings, we expected oxytocin to rise during the course of pregnancy, and indeed, 73.2% of women showed an increase in oxytocin levels from the first to the third trimester. The measurement obtained in the third trimester was close to the time of delivery and could reflect the preparation of the body for labor. In animal studies, the rise observed occurs over a very short period and is very close to the beginning of labor. Whether the same pattern occurs in some humans cannot be determined by the present study. The decrease observed after delivery was also expected as the number of oxytocin receptors decreases in the uterus of postpartum rats (30), suggesting that oxytocin is no longer playing the same physiological role as at birth.

It is unclear why some authors have found stable levels across pregnancy. As mentioned earlier, oxytocin has a very short half-life and is found in comparatively low concentrations, which makes it difficult to measure in blood (1). More importantly, the pulsatile pattern of oxytocin release (31, 32) might explain the variations from one study to another depending on when blood was drawn. However, in two studies (33, 34) where blood samples were taken two or three times and oxytocin levels averaged to try and cancel this pulsatile effect, the variations across participants remained very high, suggesting that this might not be the source of the disparate results in the literature. The large within-subject fluctuations of oxytocin levels from one time point to the other might be partly due to random variation. Thus, a possible limitation to the present study is some misclassification of women into the four categories of oxytocin (Figure 2). Finally, very little is known about factors that influence plasma oxytocin levels. Another limitation of the present study is that other variables might have affected oxytocin levels, such as mothers’ stress, sexuality, environment, or medications. On a molecular level, the amount of freely available oxytocin measured by the antibody-based assay might vary due to the amount of albumin circulating, a protein that is likely to bind oxytocin. As albumin levels decrease as pregnancy progresses (35), more oxytocin becomes available for the assay. This mechanism could explain how oxytocin levels rise during pregnancy but also brings new factors (those affecting albumin levels) into play. In parallel, in addition to its role in pregnancy, oxytocin has been shown to have significant social functions both in animals and humans (36). It is possible that oxytocin levels during the experimental visits reflect in part the qualities of the social interactions that took place with the research assistant and the nurse rather than birth variables.

Concerning the relationship between oxytocin levels and the other variables, it is important to keep in mind that all analyses were exploratory and are thus to be considered carefully, as they need to be replicated. In general, the women who were pregnant with their first child had higher oxytocin levels in the third trimester and showed a larger increase in oxytocin levels from the first to the third trimester compared to the mothers who already had one or more children. Parity also predicted the duration of labor as well as the quantity of intrapartum oxytocin administered to the mothers. The association of parity with the length of labor has long been known (37, 38). Presumably, the chances of receiving intrapartum oxytocin to accelerate labor increase with the length of labor.

While there was no direct association between oxytocin levels and the duration of labor, the fact that first-time mothers underwent a greater rise in oxytocin from the first to the third trimester and exhibited higher levels of oxytocin in the third trimester may have a functional significance. Oxytocin may mediate some of the physiological changes that are required to give birth for the first time, and these changes may leave the body permanently sensitized to oxytocin. Subsequent pregnancies may thus require a smaller increase in oxytocin.

Four additional findings emerge from this study. The first is that both the increase in oxytocin levels from the first to the third trimester, as well as the levels of oxytocin at both of those times, predicted the probability of epidural use during delivery. The second finding is that high oxytocin levels at the third trimester of pregnancy predicted a negative experience of labor. Taken together, these findings highlight the possibility of using oxytocin levels and their variations to predict some of the features of labor.

The third finding is that women who had stopped breastfeeding when interviewed at 2 months postpartum had higher oxytocin levels than those who were still breastfeeding. This result is at odds with previous reports, where higher plasma oxytocin levels were observed for breastfeeding women compared to non-breastfeeding women (39, 40). It is important to note that only slightly more than 10% of the mothers had stopped breastfeeding at 2 months postpartum. Future studies with a large number of non-breastfeeding mothers and investigating this specific question could help reconcile our findings with those of previous reports.

The last finding is that the quantity of intrapartum exogenous oxytocin administered just before delivery predicted oxytocin levels at 2 months postpartum. This is contrary to Jonas and colleagues’ findings (41) of a negative correlation between exogenous oxytocin administered during labor and plasma oxytocin levels 2 days postpartum during a breastfeeding session. However, their groups were small compared to those of the present study, and plasma oxytocin was measured 2 days postpartum, which makes our respective findings difficult to compare. In addition, Jonas and colleagues included the quantity of oxytocin administered after delivery in their analysis which we did not, because of the unreliability of these data in our sample. In any case, these findings suggest the presence of a long-term effect of intrapartum exogenous oxytocin administration on endogenous circulating oxytocin levels. Little is known about other possible long-term effects of oxytocin administration during labor on the physiology of the mother. A recent study (42), however, has linked intrapartum exogenous oxytocin to difficulties with sucking behavior in the newborn and a decreased duration of breastfeeding. Given the role of oxytocin in lactation, the long-term effect of exogenous oxytocin on endogenous oxytocin may be relevant to explaining this finding. In addition, it remains unknown whether exogenous oxytocin administered during labor affects the fetus and, if so, how. Further, a number of studies have shown that exogenous oxytocin produces significant psychological effects, including improved self-perception (43), improved perception of social relationships (44), decreased memory for objects visually perceived (45), and significant physiological effects including on the immune system (19). It is possible, therefore, that exogenous oxytocin affects the psychological and physiological states of the mother or, indeed, of the newborn. Many women giving birth in North America receive intrapartum oxytocin to induce or accelerate labor. Our finding of a direct association between exogenous oxytocin administered before delivery and the levels of plasma oxytocin 2 months postpartum provide evidence of a possible long-term effect of this routine intervention, the consequences of which are largely unknown. Whether oxytocin administration, either during labor or following delivery, has other physiological or psychological effects, and whether those effects persist, are questions of some urgency for future research.

## Conflict of Interest Statement

Togas Tulandi is a clinical investigator funded by HALT Medical and advisor for Watson Pharma. All other authors declare that the research was conducted in the absence of any commercial or financial relationships that could be construed as a potential conflict of interest.
